# STRONGER TOGETHER: GLOBAL COMPARISONS OF THE USE OF CITIZEN SCIENCE TO CREATE AGE- AND DEMENTIA-FRIENDLY COMMUNITIES

**DOI:** 10.1093/geroni/igad104.1040

**Published:** 2023-12-21

**Authors:** Annie Robitaille, Kimberly Campbell, Linda Garcia

## Abstract

The active involvement of older people in the development of age-friendly environments has been strongly recommended. However, participatory methods with older adults remain underutilised even though older people may be more qualified to recommend what makes an age-friendly space. Using a citizen science approach in research involving older adults presents a key opportunity to have them be active participants and for researchers to obtain meaningful insight. Using this approach, older adults are viewed as co-designers. They are involved in the research process from beginning to end - research design, development of tools, data collection, analysis and knowledge dissemination. This symposium will highlight some of the work done in Canada, Australia and the United Kingdom to make communities more age-friendly using a citizen science approach. Special attention will be given to how the approach might be adapted to citizen scientists who live with dementia. After an introductory overview of the citizen science approach, the use of this method in studies including persons living with dementia and their care partners will be discussed, both in general terms and as an application to air travel. One of the presentations will also address the use of citizen science in examining population ageing and urbanization. Our symposium will conclude with a discussion of future policy, practice, and research direction.

